# Parallel and deep reservoir computing using semiconductor lasers with optical feedback

**DOI:** 10.1515/nanoph-2022-0440

**Published:** 2022-10-17

**Authors:** Hiroshi Hasegawa, Kazutaka Kanno, Atsushi Uchida

**Affiliations:** Department of Information and Computer Sciences, Saitama University, 255 Shimo-okubo, Sakura-ku, Saitama City, Saitama 338-8570, Japan

**Keywords:** deep learning, machine learning, semiconductor laser, time delay

## Abstract

Photonic reservoir computing has been intensively investigated to solve machine learning tasks effectively. A simple learning procedure of output weights is used for reservoir computing. However, the lack of training of input-node and inter-node connection weights limits the performance of reservoir computing. The use of multiple reservoirs can be a solution to overcome this limitation of reservoir computing. In this study, we investigate parallel and deep configurations of delay-based all-optical reservoir computing using semiconductor lasers with optical feedback by combining multiple reservoirs to improve the performance of reservoir computing. Furthermore, we propose a hybrid configuration to maximize the benefits of parallel and deep reservoirs. We perform the chaotic time-series prediction task, nonlinear channel equalization task, and memory capacity measurement. Then, we compare the performance of single, parallel, deep, and hybrid reservoir configurations. We find that deep reservoirs are suitable for a chaotic time-series prediction task, whereas parallel reservoirs are suitable for a nonlinear channel equalization task. Hybrid reservoirs outperform other configurations for all three tasks. We further optimize the number of reservoirs for each reservoir configuration. Multiple reservoirs show great potential for the improvement of reservoir computing, which in turn can be applied for high-performance edge computing.

## 1 Introduction

Recent photonics technologies, such as wavelength multiplexing and photonic integrated circuits, have enabled high-speed and energy-efficient signal processing in the field of communication and computation. These photonics technologies can overcome the limitation in the development of semiconductor integration technologies known as the end of Moore’s law [1]. Photonic hardware accelerators have been intensively investigated to improve the performance of signal processing in machine learning tasks [1]. Several examples of photonics accelerators include photonic neural networks [2] for image recognition, coherent Ising machine [3] for solving a max-cut problem, photonic decision making [4] for solving a reinforcement-learning problem, and photonic reservoir computing [5, 6] for time-series prediction and speech recognition.

There has been a considerable rise in the demand and subsequent interest in reservoir computing in the past two decades [7, 8]. The conceptual idea of reservoir computing originated from a recurrent neural network with randomly fixed weights of the input-node and inter-node connections. A simple learning approach, such as the least-square method, can be applied for the connection weights between network nodes and output (i.e., readout) for the ease of the implementation of reservoir computing. The introduction of reservoir computing has led to the utilization of many physical devices such as spintronics, nanodevices, electronics, and photonics, as reservoirs [9]. Photonic reservoir computing is mainly implemented in spatial optical systems (spatial reservoirs) and time-delayed optical systems (delay-based reservoirs). In spatial reservoirs, network nodes in a reservoir are constructed in space using a spatial light modulator [10, 11], passive optical array components [12], and a large-area vertical-cavity surface-emitting laser [13]. Spatial nodes are used to calculate the weighted linear sum of the node states for the output signal. In contrast, in time-delayed reservoirs, a single nonlinear optical component with a time-delayed feedback loop is used as a reservoir by integrating a semiconductor laser [14–16], semiconductor optical amplifier [17], and Mach–Zehnder electro-optic modulator [5, 18]. Network nodes are measured in time, and virtual nodes are measured by sampling the temporal waveform of the reservoir output. A weighted linear sum of the virtual node states is used to obtain the output signal. The primary benefits of delay-based reservoirs include easy implementation and large-scale network construction based on an increase in the delay time, whereas those of spatial reservoirs include real-time implementation without pre- and post-processing.

The ease in implementation enables the wide use of signal processing in reservoir computing. However, the lack of optimization of inter-node and input-node connection weights limits the performance of signal processing in reservoir computing because these weights are randomly fixed in advance. To overcome this issue, parallel and deep configurations of multiple reservoirs have been proposed to improve the overall performance of reservoir computing [19]. An example of such a configuration is the parallel reservoir, wherein semiconductor lasers with short external cavities are used, and the performance of chaotic time-series prediction tasks is improved by increasing the number of parallel reservoirs [20]. Several configurations of parallel reservoirs have been proposed in time-delayed systems [21] and mutually coupled vertical-cavity surface-emitting lasers [22]. Parallel reservoirs have also been used for solving multiple tasks in parallel [23, 24]. In addition, deep (serial) reservoirs have been proposed in time-delayed optoelectronic systems [25], and the memory capacity has been evaluated for a different number of layers. Moreover, various multiple reservoir configurations have been evaluated using spatial passive photonic circuits with interferometers [26], and the use of multiple reservoirs may help improve the performance of a header recognition task. However, deep configuration using delay-based all-optical reservoirs has not been investigated yet, although a delay-based all-optical reservoir has been experimentally implemented in a photonic integrated circuit using a semiconductor laser with optical feedback for high-speed processing [16]. Furthermore, a quantitative comparison between parallel and deep configurations with delay-based all-optical reservoirs has not been performed, and the dependence of the performance using different reservoir configurations on the types of signal-processing tasks has not been clearly studied.

In this study, we propose single, parallel, deep, and hybrid reservoir configurations using semiconductor lasers with optical feedback to improve the performance of time-delayed all-optical reservoir computing. We compare the performance of these reservoir configurations using different tasks: chaotic time-series prediction task, nonlinear channel equalization task, and memory capacity measurement. We also optimize the number of multiple reservoirs using these configurations and signal-processing tasks.

## 2 Methods

### 2.1 Numerical model of a semiconductor laser with optical feedback

**Figure 1:**
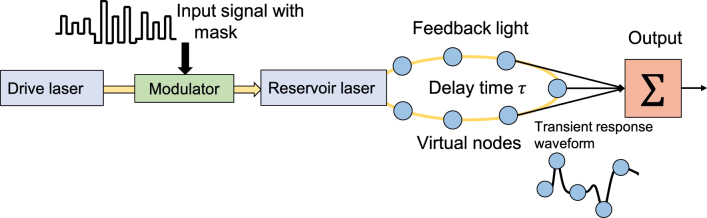
Reservoir computing using a semiconductor laser with optical feedback and injection.

Figure 1 shows the schematic of delay-based all-optical reservoir computing using a semiconductor laser with optical feedback and injection [15]. We define all-optical reservoirs as the reservoirs that consist of all-optical devices without considering pre- and post-processing for delay-based reservoir computing. A semiconductor laser (referred to as a reservoir laser) with optical feedback is used as a photonic reservoir. The reservoir laser comprises an optical feedback loop with a delay time *τ*. The light intensity from another semiconductor laser (referred to as a drive laser) is modulated using an input signal with a random binary mask signal. The modulated light is injected into the reservoir laser. The temporal waveform of the output of the reservoir laser is sampled at an interval *θ*, and the sampled data are considered as virtual nodes in the time-delayed feedback loop. The number of virtual nodes is determined using the equation *N* = *τ*/*θ*. A weighted linear sum of the virtual node states is calculated as the output signal. The output weights of the virtual node states are trained using the linear least-squares method such that they match the output signal with the original target signal.

The dynamics of the reservoir laser with optical feedback and injection can be described using the Lang–Kobayashi equations as [27, 28],

(1) $$\begin{array}{ll} \frac{dE_{r}\left(t\right)}{dt} & =\frac{1+i\alpha }{2}\left\{\frac{G_{N}\left(N_{r}\left(t\right)-N_{0}\right)}{1+\varepsilon {\left|E_{r}\left(t\right)\right|}^{2}}-\frac{1}{\tau_{p}}\right\}E_{r}\left(t\right)\\ & \;+\kappa E_{r}\left(t-\tau \right)\exp\left(-i\omega_{r}\tau \right)\\ & \;+\kappa_{\text{inj}}E_{d}\left(t\right)\exp\left(i2\pi \Delta ft\right)+\xi \left(t\right) \end{array}$$

(2) $$\frac{dN_{r}\left(t\right)}{dt}=J-\frac{N_{r}\left(t\right)}{\tau_{s}}-\left\{\frac{G_{N}(N_{r}\left(t\right)-N_{0}}{1+\varepsilon {\left|E_{r}\left(t\right)\right|}^{2}}\right\}{\left|E_{r}\left(t\right)\right|}^{2}$$

where *E*_*r*_(*t*) and *N*_*r*_(*t*) represent the complex electric-field amplitude and the carrier density of the reservoir laser, respectively. Here, *G*_*N*_ is the gain coefficient, *κ* is the feedback strength, *κ*_inj_ is the injection strength, *Δf* is the optical frequency detuning between the drive and reservoir lasers, *J* is the injection current, *ε* is the gain saturation coefficient, and *ξ*(*t*) is the spontaneous emission noise. The optical intensity *I*_*r*_(*t*) of the reservoir laser is expressed as *I*_*r*_(*t*) = |*E*_*r*_(*t*)|^2^. The electric-field amplitude of the phase-modulated drive laser is expressed as [29],

(3) $$E_{d}\left(t\right)=\sqrt{I_{d}}\exp\left(i\pi u\left(t\right)\right)$$

where *I*_*d*_ is the constant optical intensity of the drive laser and *u*(*t*) is the modulation signal with the input signal. The laser parameters of all reservoirs used in our numerical simulations are summarized in Table 1.

**Table 1:** Parameter values of the reservoir laser used in numerical simulations.

Symbol	Parameter	Value
*G*_*N*_	Gain coefficient	8.40 × 10^−13^ m^3^ s^−1^
*N*_0_	Carrier density at transparency	1.40 × 10^24^ m^−3^
*ε*	Gain saturation coefficient	2.0 × 10^−23^
*τ*_*p*_	Photon lifetime	1.927 × 10^−12^ s
*τ*_*s*_	Carrier lifetime	2.04 × 10^−9^ s
*α*	Linewidth enhancement factor	3.0
*τ*	Feedback delay time of light	8.04 × 10^−8^ s
*r*_*3*_	Reflectivity of external mirror	0.10
*κ*	Feedback strength	15.53 × 10^9^ s^−1^
*κ*_inj_	Injection strength from drive laser	12.42 × 10^9^ s^−1^
*ω =* 2*πc/λ*	Optical angular frequency	1.226 × 10^15^ s^−1^
*λ*	Optical wavelength	1.537 × 10^−6^ m
*c*	Speed of light	2.998 × 10^8^ m s^−1^
*J*_*d*_	Injection current of drive laser	1.30 *J*_th_
*J*_*r*_	Injection current of reservoir laser	1.05 *J*_th_
*J*_th_ *= N*_th_*/τ*_*s*_	Injection current at lasing threshold	9.892 × 10^32^ m^−3^ s^−1^
*N*_th_ *= N*_0_ *+* 1*/G*_*N*_*τ*_*p*_	Carrier density at lasing threshold	2.018 × 10^24^ m^−3^
Δ*f*	Initial optical frequency detuning	−3.0 × 10^9^ Hz

We do not use the ridge regression procedure for training. Instead, we add spontaneous emission noise to the reservoir laser, which plays a similar role to ridge regression, and the performance of reservoir computing can be improved in the presence of noise [30].

### 2.2 Configurations of parallel and deep reservoir computing

**Figure 2:**
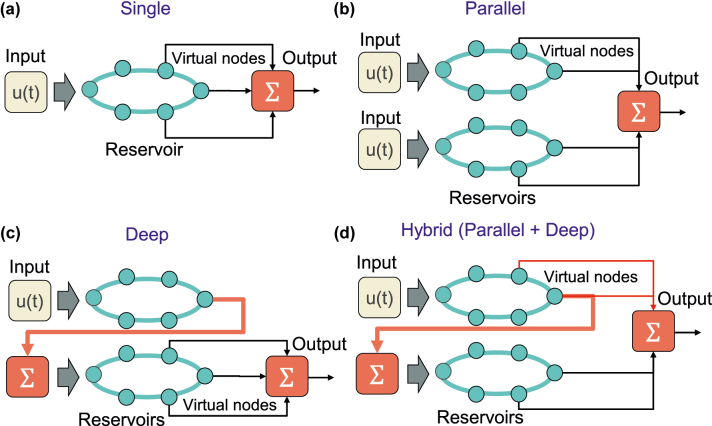
Configurations of parallel and deep reservoir computing. (a) Single reservoir, (b) parallel reservoirs, (c) deep reservoirs, and (d) hybrid reservoirs.

We propose four different reservoir configurations to evaluate the impact of parallel and deep (serial) reservoir computing. Figure 2 shows the diagrams of the four reservoir configurations. Figure 2(a) shows a single reservoir that consists of a single semiconductor laser with optical feedback. The input signal is injected into the reservoir laser, and the output is calculated from a weighted linear sum of the virtual node states in the reservoir output. The total number of virtual nodes *N*_total_ matches the number of virtual nodes in the single reservoir *N* (*N*_total_ = *N*).

Figure 2(b) shows parallel reservoirs, wherein multiple reservoirs are configured in parallel. The same input signal with a different mask signal is injected into each reservoir to obtain different dynamics of each reservoir output. The virtual node states of all parallel reservoirs are used to generate the output signal by calculating a weighted linear sum of the virtual node states. Thus, the total number of virtual node states is given as *N*_total_ = *kN* for *k* reservoirs and the number of virtual nodes for each reservoir *N*.

Figure 2(c) shows deep reservoirs, wherein multiple reservoirs are cascaded in serial. The input signal with a mask signal is injected into the first reservoir. A weighted linear sum of the virtual node states of the first reservoir is calculated as the output of the first reservoir. The output of the first reservoir is then used as an input signal for the second reservoir. The output of the first reservoir with a different mask signal is injected into the second reservoir. A weighted linear sum of the virtual node states of the second reservoir is calculated to generate the output signal, which is further used as an input signal for the third reservoir. This sequential procedure is repeated, and a weighted linear sum of the final reservoir is considered as the output of the entire deep reservoir. The total number of virtual node states is given as *N*_total_ = *kN*. However, the final output is obtained only from *N* virtual node states from the *k*th (final) reservoir.

In deep reservoirs, output weights for each reservoir are trained such that the difference between each reservoir output and the original target signal is minimized using the linear least-squares method. The target signal used in the training of all reservoirs is the same; however, the input signal to each reservoir is different because the output signal of the *i*th reservoir (i.e., the weighted linear sum of the *i*th reservoir nodes) is used as the input signal of the (*i* + 1)-th reservoir for the deep configuration. If the prediction error is small at the first reservoir, the input signal of the second reservoir already resembles the target signal, and the prediction error at the first reservoir can be easily compensated by the second reservoir.

Furthermore, we propose a hybrid configuration for parallel and deep reservoirs. Figure 2(d) shows hybrid reservoirs, wherein the structure of multiple reservoirs is the same as that of deep reservoirs shown in Figure 2(c). The input signal with a mask signal is injected into the first reservoir only, and the output of the first reservoir (the weighted linear sum of the virtual node states in the first reservoir) is used as the input signal for the second reservoir. This procedure is repeated for the entire configuration. However, virtual node states of all multiple reservoirs are used to generate the final output signal, and this is similar to that of parallel reservoirs shown in Figure 2(b). The total number of virtual node states is given as *N*_total_ = *kN*, and the final output is also obtained using *N*_total_ virtual node states from all multiple reservoirs.

## 3 Numerical results

### 3.1 Chaotic time-series prediction task

We compare the performance of the four reservoir configurations shown in Figure 2. We first use the Santa Fe chaotic time-series prediction task [31] to evaluate the performance of the four reservoir configurations. The aim of this prediction task is to perform single-point prediction of chaotic data that is generated from a far-infrared laser. Here, 3000 steps are used for training and 1000 steps are used for testing. The amplitude of the chaotic time series for prediction is normalized, and the input signal *u*(*t*) of the chaotic time series ranges from 0 to 1.

We introduce a quantitative measure for performance evaluation. The normalized mean square error (NMSE) is defined as follows:

(4) $$NMSE=\frac{1}{L}\frac{\overset{L}{\underset{n=1}{\sum }}{\left(\bar{y}\left(n\right)-y\left(n\right)\right)}^{2}}{var\left(\bar{y}\right)}$$

where *n* is the index of the input data, *L* is the total number of datasets, *y* is the reservoir output that is compared with the original value *y*¯ as a target, and var represents the variance.

In this section, we evaluate the configurations with two reservoirs (*k* = 2) in parallel, deep, and hybrid reservoirs, as shown in Figure 2. The period of the mask signal is set as *T* = 80.4 ns. The feedback delay time and sampling interval are set as *τ* = 80.4 ns and *θ* = 0.1 ns, respectively. Therefore, the number of virtual node states for each reservoir is set as *N* = 800 (the four remaining nodes are discarded).

**Figure 3:**
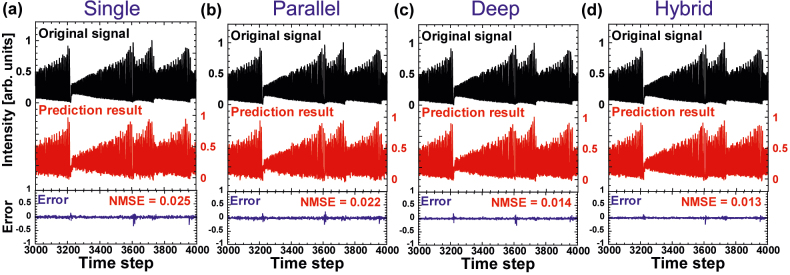
Results of the chaotic time-series prediction task using (a) single, (b) parallel, (c) deep, and (d) hybrid configurations. The original target signal (black), prediction result (red), and error between them (blue) are shown.

Figure 3 shows the results of the chaotic time-series prediction task for single, parallel, deep, and hybrid reservoirs. The black, red, and blue curves represent the original target signal, prediction result, and error signal between them, respectively. For all cases, the prediction result resembles the original target signal, and the error signal is extremely small. However, a difference in the errors appears when NMSE values are calculated. The NMSE values for single, parallel, deep, and hybrid reservoirs are 0.025, 0.022, 0.014, and 0.013, respectively. Therefore, the performance of deep and hybrid reservoirs is better (smaller errors) than that of single and parallel reservoirs, as shown in Figure 3.

**Figure 4:**
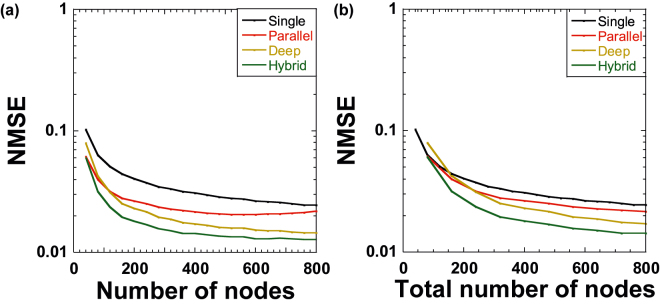
Normalized mean-square error (NMSE) of the chaotic time-series prediction task as a function of (a) the number of nodes *N* for each reservoir and (b) the total number of nodes *N*_total_ for all reservoirs for single (black), parallel (red), deep (brown), and hybrid (green) configurations. (a) *N*_total_ is *N* for single configuration and 2*N* for parallel, deep, and hybrid configurations. (b) *N*_total_ is matched to *N* for all configurations.

We systematically compare the performance of the four reservoir configurations in the chaotic time-series prediction task when the number of nodes *N* for each reservoir is changed. We change the value of *N* up to 800 by discarding the virtual node states without changing the values of *τ* and *θ*. We use the first *N* virtual nodes in the mask period *T*. Figure 4(a) shows the results of the prediction error (NMSE) as *N* is changed for single, parallel, deep, and hybrid reservoirs. The hybrid reservoir shows the smallest NMSE values for different values of *N* among the four configurations, and the deep reservoir demonstrates the second-best performance. Although the performance of the parallel reservoir is worse than that of hybrid and deep reservoirs, it is better than that of the single reservoir. From these results, we suggest that hybrid and deep configurations with serial reservoir connections are effective for the chaotic time-series prediction task.

In Figure 4(a), the total number of virtual nodes *N*_total_ is different for each configuration, i.e., the total number of nodes is *N* for the single reservoir and 2*N* for parallel, deep, and hybrid configurations with two reservoirs. Here, we match *N*_total_ to *N* to suppress the impact of the difference in *N*_total_. For instance, one-half of the number of nodes is used for parallel, deep, and hybrid reservoirs by discarding the remaining virtual nodes. This ensures that the total number of nodes becomes *N*_total_ = *N* for all four configurations without changing the values of *τ* and *θ*.

Figure 4(b) shows the NMSE values for the prediction task as *N*_total_ is changed for the four configurations. The result of the performance comparison is similar to that shown in Figure 4(a), that is, the order of the best to worst performance is hybrid, deep, parallel, and single reservoirs. Therefore, the number of nodes is not sufficiently effective for a comparison in this case. Notably, NMSE values are similar to each other when *N*_total_ is small (around 100), and the difference in NMSE values is apparent for a large *N*_total_ (around 800), as shown in Figure 4(b).

In the deep configuration, the first reservoir predicts the original target signal by learning. Some prediction errors may be observed between the target signal and the output of the first reservoir. These errors can be compensated by the second reservoir, primarily because the second reservoir is trained for eliminating these prediction errors through learning. In other words, the error correction of the predicted signal from the first reservoir can be achieved using the second reservoir. Therefore, the first reservoir roughly predicts the target signal, and the second reservoir corrects the prediction errors for a more accurate prediction. The deep configuration is thus suitable for the time-series prediction task. In addition, the hybrid configuration has a similar reservoir structure to the deep configuration and is also suitable for this task.

### 3.2 Nonlinear channel equalization task

Next, we use the nonlinear channel equalization task [18] to compare the performance of the four configurations. The purpose of the nonlinear channel equalization task is to classify the four digital signals {−3, −1, 1, and 3} transmitted through a communication channel with nonlinear distortion. The nonlinear transformation of the communication channel is described as follows:

(5) $$\begin{array}{ll} q\left(n\right) & =0.08d\left(n+2\right)-0.12d\left(n+1\right)+d\left(n\right)+0.18d\left(n-1\right)\\ & \;-0.1d\left(n-2\right)+0.091d\left(n-3\right)-0.05d\left(n-4\right)\\ & \;+0.04d\left(n-5\right)+0.03d\left(n-6\right)+0.01d\left(n-7\right) \end{array}$$

(6) $$u\left(n\right)=q\left(n\right)+0.036q{\left(n\right)}^{2}-0.011q{\left(n\right)}^{3}+v\left(n\right)$$

where *d*(*n*) is the input signal of a random sequence with values {−3, −1, +1, +3}, *q*(*n*) is the linear channel output, *u*(*n*) is the noisy nonlinear channel output, and *v*(*n*) is the white Gaussian noise with a zero mean to yield signal-to-noise ratios (SNRs). The term *u*(*n*) is used to determine *d*(*n*) using reservoir computing. A symbol error rate (SER) is used to evaluate the performance of this task, and a smaller SER value indicates better performance.

**Figure 5:**
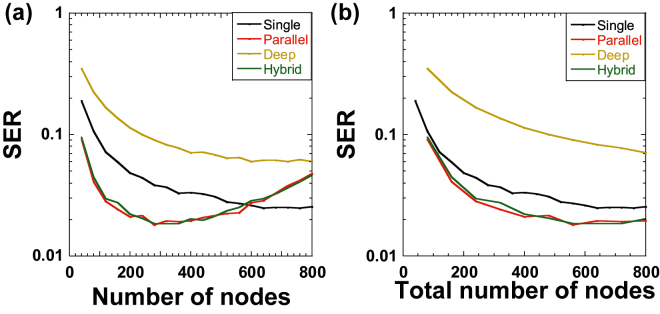
Symbol error rate (SER) of the nonlinear channel equalization task as a function of (a) number of nodes *N* for each reservoir and (b) the total number of nodes *N*_total_ for all reservoirs for single (black), parallel (red), deep (brown), and hybrid (green) configurations. (a) *N*_total_ is *N* for the single configuration and 2*N* for parallel, deep, and hybrid configurations. (b) *N*_total_ is matched to *N* for all four configurations.

Figure 5(a) shows the results of the nonlinear channel equalization task as the number of nodes *N* for each reservoir is changed in each configuration. In this case, the total number of nodes is *N*_total_ = *N* for the single reservoir and *N*_total_ = 2*N* for parallel, deep, and hybrid reservoirs. Lower SER values are obtained for hybrid and parallel reservoirs, and, therefore, better performance is achieved for these configurations. A minimum SER value of 0.018 is obtained at *N* = 280 for parallel and hybrid reservoirs, as shown in Figure 5(a). However, the value of SER increases as *N* is increased above 400 for parallel and hybrid reservoirs because too many node states may result in overtraining. In addition, the SER value for the deep reservoir is worse than that for the single reservoir. This indicates that the deep configuration is not suitable for the nonlinear channel equalization task.

Figure 5(b) shows the result of SER in the nonlinear channel equalization task when *N*_total_ is matched among the four configurations and *N*_total_ is changed instead of *N*. Parallel and hybrid reservoirs outperform single and deep reservoirs. In addition, the SER value for the deep reservoir is the worst among the four configurations. Therefore, multiple reservoirs do not always provide better performance in this task.

The nonlinear channel equalization task requires a four-digit classification from a distorted analog signal with nonlinearity and noise. The second reservoir of the deep configuration helps correct the errors between the target signal and the output of the first reservoir. However, the errors may be enhanced owing to the discretization of the output signal for the four-digit classification and cannot be compensated by the second reservoir, unlike the time-series prediction task. Therefore, the deep configuration is not appropriate for the nonlinear channel equalization task. In contrast, the parallel configuration provides multiple reservoirs with different output weights that are trained using the same input signal with different mask signals. Therefore, we consider that the generalization ability may be enhanced using parallel reservoirs. From Figures 4 and 5, we found that suitable reservoir configurations depend on the type of processing tasks.

### 3.3 Memory capacity

We also investigate the memory capacity of all four configurations. Memory capacity is a measure of the amount of information of past input signals that can be reproduced through reservoir computing [32, 33]. Memory capacity is defined using the correlation function *m*(*i*),

(7) $$m\left(i\right)=\frac{{\left\langle \left(y\left(n-i\right)\right)\left(o_{i}\left(n\right)\right)\right\rangle }^{2}}{\sigma^{2}\left(y\left(n\right)\right)\sigma^{2}\left(o_{i}\left(n\right)\right)}$$

where *y*(*n*) is a random input signal in the range from −1 to 1, *o*_*i*_(*n*) is the reservoir output at time *n* when the output weights are trained with the *i*th past input signal *y*(*n* − *i*), *σ*^2^ is the variance, and <> denotes the time average. Memory capacity is described as the sum of *m*(*i*) by,

(8) $$MC=\overset{\infty }{\underset{i=1}{\sum }}m\left(i\right)$$

A higher memory capacity value indicates a better reservoir for the tasks that require previous information.

**Figure 6:**
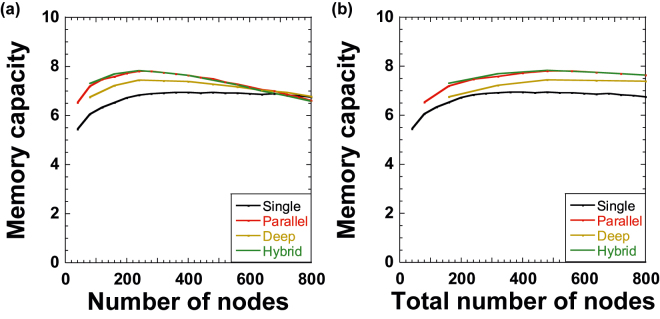
Memory capacity as a function of (a) the number of nodes *N* for each reservoir and (b) the total number of nodes *N*_total_ for all reservoirs for single (black), parallel (red), deep (brown), and hybrid (green) configurations. (a) *N*_total_ is *N* for the single configuration and 2*N* for parallel, deep, and hybrid configurations. (b) *N*_total_ is matched to *N* for all four configurations.

Figure 6(a) shows the memory capacity when *N* is changed for the four configurations. The memory capacity of parallel and hybrid reservoirs is larger than that of single and deep reservoirs. In addition, an optimal memory capacity of 7.8 is obtained at *N* = 280 for parallel and hybrid reservoirs, as shown in Figure 6(a). However, memory capacity is almost the same at *N* = 800 among the four configurations.

Figure 6(b) shows the memory capacity when *N*_total_ is matched among the four configurations and *N*_total_ is changed. Parallel and hybrid reservoirs provide a larger memory capacity compared with deep reservoirs, and the memory capacity of deep reservoirs is better than that of single reservoirs. However, there is no significant difference in memory capacity among the four configurations. The memory capacity values are 6.61, 7.65, 7.38, and 7.64 for single, parallel, deep, and hybrid reservoirs at *N*_total_ = 800, respectively.

Multiple reservoirs provide a larger memory capacity than a single reservoir because we speculate that an input signal is stored in multiple reservoirs with different mask signals. However, the difference in the memory capacity is not significant among parallel, deep, and hybrid reservoirs. Therefore, memory capacity does not strongly depend on the type of reservoir configurations.

The best evaluation values for the three aforementioned tasks in the four reservoir configurations are shown in Table 2. The total number of nodes is fixed at *N*_total_ = 800 to avoid dependence on the number of nodes shown in Table 2. For the chaotic time-series prediction task, the minimum NMSE value (0.016) is obtained using the hybrid reservoir, and the deep reservoir shows the second-best performance (NMSE = 0.017). On the contrary, for the nonlinear channel equalization task, parallel and hybrid reservoirs provide the best evaluation value (SER = 0.020). Regarding the memory capacity, the parallel reservoir shows the best performance (MC = 7.65) followed by the hybrid reservoir (MC = 7.64). However, there is a small difference in the memory capacity of parallel, deep, and hybrid reservoirs.

**Table 2:** Comparison of the best performance among single, parallel, deep, and hybrid configurations in the chaotic time-series prediction task, nonlinear channel equalization task, and memory capacity measurement. The total number of nodes is *N*_total_ = 800.

Configuration	Chaotic time-series prediction task (NMSE)	Nonlinear channel equalization task (SER)	Memory capacity (MC)
Single	0.025	0.025	6.61
Parallel	0.022	0.020	7.65
Deep	0.017	0.072	7.38
Hybrid	0.016	0.020	7.64

From these results, we observe that deep configuration (serial reservoir connection) is suitable for the chaotic time-series prediction task, whereas parallel configuration (all node states are used for the final output) is suitable for the nonlinear channel equalization task. The hybrid reservoir, which possesses the characteristics of both deep and parallel configurations, shows the best performance for all three tasks. Therefore, the hybrid reservoir outperforms the other three reservoirs for different types of tasks. For hybrid reservoirs, the deep configuration is beneficial for correcting prediction errors in the time-series prediction task, and the parallel configuration that uses all node states for calculating the output signal is suitable for the nonlinear channel equalization task.

## 4 Effect of the number of reservoirs

### 4.1 Chaotic time-series prediction task

In the previous section, we considered two reservoirs for each configuration to evaluate their performance. In this section, we investigate the impact of the number of reservoirs on the chaotic time-series prediction task. We change the number of reservoirs *k* and find the optimal value of *k* for parallel, deep, and hybrid reservoir configurations. To increase the number of nodes, the period of the mask signal is set as *T* = 260.5 ns. The feedback delay time and sampling interval are set as *τ* = 260.5 ns and *θ* = 0.1 ns, respectively. Therefore, the maximum number of nodes is *N* = 2605, and the value of *N* is changed by discarding the remaining nodes.

**Figure 7:**
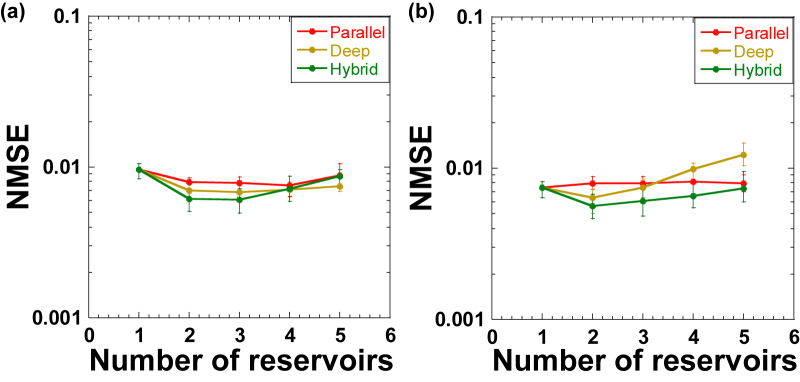
Normalized mean-square error (NMSE) of the chaotic time-series prediction task as the number of reservoirs *k* is changed for parallel (red), deep (brown), and hybrid (green) configurations. (a) The number of nodes for each reservoir is fixed at *N* = 280. (b) The total number of nodes is fixed at *N*_total_ = 720.

Figure 7(a) shows the prediction error (NMSE) of the chaotic time-series prediction task when *k* is changed for parallel, deep, and hybrid configurations. In this case, the number of nodes for each reservoir is fixed at *N* = 280, and the total number of nodes *N*_total_ = *kN* increases with *k*. As shown in Figure 7(a), for parallel reservoirs, the NMSE value slightly decreases as the *k* value increases, and the minimum NMSE value is obtained when *k* = 4. In contrast, for deep and hybrid reservoirs, the minimum NMSE value is obtained when *k* = 3, which is 0.0060. Here, the value of *N*_total_ increases, and the performance is improved when *k* is increased up to 3; however, too many nodes may result in overtraining. The optimal *k* value for the three configurations is obtained in Figure 7(a).

To suppress the effect of the change in *N*_total_, we fixed *N*_total_ when *k* is changed. Figure 7(b) shows the prediction error (NMSE) for the chaotic time-series prediction task when *k* is changed under the condition of a fixed *N*_total_ = *kN* = 720. For instance, *N* is set at 360 for *k* = 2, *N* = 240 is used for *k* = 3, and so on; here, the remaining nodes at the fixed *τ* and *θ* are discarded. In this case, for parallel reservoirs, NMSE values are almost unchanged for different values of *k*. This indicates that the value of *N*_total_ has a significant impact on the performance. However, the division into smaller reservoirs in parallel is not effective at a constant *N*_total_ for parallel reservoirs, especially for this task. On the contrary, for deep and hybrid reservoirs, the minimum NMSE value is obtained when *k* = 2 (i.e., an NMSE value of 0.0056 for hybrid reservoirs). A larger *k* value increases the NMSE value as *N* is decreased for each reservoir. This indicates that two reservoirs are sufficient for deep and hybrid reservoirs in this task. We interpret that the first reservoir predicts the input signal, and the second reservoir corrects the errors between the target signal and the predicted signal in the first reservoir for the deep configuration. The roles of the third and more reservoirs are similar to that of the second reservoir (i.e., error correction of the predicted signal), and they cannot effectively improve the performance of the time-series prediction task.

From these results, the use of three reservoirs provides the best performance for parallel, deep, and hybrid reservoirs when *N*_total_ is changed because the number of nodes increases with *k*. In contrast, the use of two reservoirs provides the best performance for deep and hybrid reservoirs when *N*_total_ is fixed. A larger number of reservoirs may result in the degradation of performance owing to the lack of the number of nodes for each reservoir when the value of *N*_total_ is fixed. In addition, the two-reservoir configuration is the best for the time-series prediction task because the second reservoir plays an effective role in error correction between the target signal and the predicted output from the first reservoir in deep and hybrid configurations.

### 4.2 Nonlinear channel equalization task

Next, we investigate the impact of the number of reservoirs on the nonlinear channel equalization task. Similar to the procedure described in Section 4.1, we change the number of reservoirs *k* and find the optimal value of *k* for parallel, deep, and hybrid reservoir configurations.

**Figure 8:**
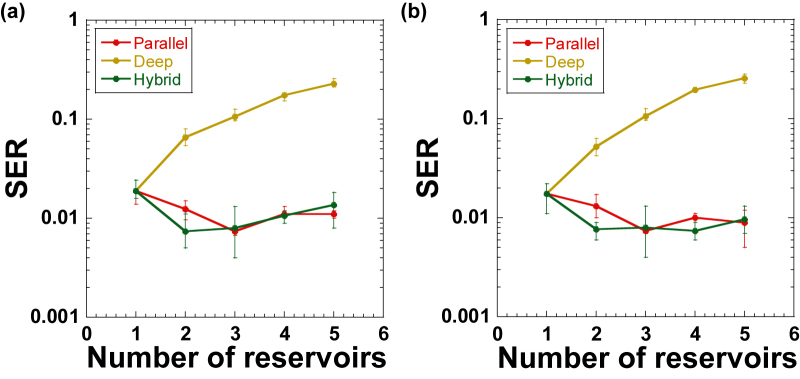
Symbol error rate (SER) of the nonlinear channel equalization task as the number of reservoirs *k* is changed for parallel (red), deep (brown), and hybrid (green) configurations. (a) The number of nodes for each reservoir is fixed at *N* = 160. (b) The total number of nodes is fixed at *N*_total_ = 480.

First, we set *N* = 160 and change the value of *k* (*N*_total_ = *kN* is also changed). Figure 8(a) shows the SER value of the nonlinear channel equalization task when *k* is changed. For deep reservoirs, the SER value increases significantly as the *k* value increases. Therefore, the deep configuration is not suitable for the nonlinear channel equalization task. For parallel and hybrid reservoirs, the SER value decreases, and a minimum SER value of 0.0073 is obtained when *k* = 3 and *k* = 2, respectively. Therefore, the performance can be improved by optimizing the *k* value for parallel and hybrid configurations.

We then set *N*_total_ = 480 instead of *N*, and change the *k* value, as shown in Figure 7(b). Figure 8(b) shows the SER value of the nonlinear channel equalization task when *k* is changed and *N*_total_ is fixed. In this case, the results are similar to those shown in Figure 8(a). The SER value for deep reservoirs is worse when the *k* value is increased. For parallel reservoirs, the minimum SER value is obtained when *k* = 3. The curve shown in Figure 8(b) for parallel reservoirs is similar to that shown in Figure 8(a), and the performance depends on the *k* value but not the number of nodes. For hybrid reservoirs, the minimum SER value is achieved when *k* = 4, although SER values are similar when *k* = 2, 3, and 4. Therefore, the performance of the nonlinear channel equalization task is improved by optimizing the *k* value for parallel and hybrid reservoirs.

From these results, three or four reservoirs are sufficient to improve the performance of the nonlinear channel equalization task for parallel and hybrid configurations. We speculate that too many reservoirs may result in a lack of the number of nodes for each reservoir. In addition, too many nodes may lead to overtraining. Therefore, optimizing the number of reservoirs is necessary for multiple reservoir configurations.

## 5 Discussion

We investigated and compared the performance of single, parallel, deep, and hybrid reservoir configurations in three different processing tasks. For the chaotic time-series prediction task, deep and hybrid reservoir configurations outperform single and parallel reservoir configurations. In deep reservoirs, the second reservoir receives the output signal of the first reservoir, and the prediction errors in this signal are corrected by the second reservoir. Therefore, the deep (serial) configuration can effectively improve the performance of the time-series prediction task. For a configuration with three or more reservoirs, the effect of error correction is minimal, and the use of two reservoirs is optimal for the deep configuration to improve the performance of the chaotic time-series prediction task.

For the nonlinear channel equalization task, parallel and hybrid reservoirs outperform single and deep reservoirs. For this task, a four-digit classification is required from analog data that are distorted by nonlinearity and noise. The second reservoir in the deep configuration cannot sufficiently correct the discretized data, and error correction cannot be achieved effectively. For the parallel configuration, classification can be achieved by using multiple reservoirs with different input masks for the same input signal. The presence of multiple reservoirs with different readout weights may enhance the performance of the classification task.

In terms of memory capacity, there is no significant difference in the performance among multiple reservoirs, although the memory capacity of multiple reservoirs is better than that of a single reservoir. The change in the configuration of multiple reservoirs cannot effectively improve the memory capacity.

Hybrid reservoirs outperform the other three reservoirs because the hybrid configuration provides both deep and parallel configurations. The use of a second reservoir in the deep configuration can help correct prediction errors between the target signal and the predicted signal of the first reservoir. In addition, the use of node states from all multiple reservoirs can effectively improve classification performance. Therefore, hybrid reservoirs are extremely useful in all three processing tasks.

Furthermore, one of the advantages of hybrid reservoirs is their wide applicability to different types of tasks. Hybrid reservoirs outperform parallel and deep reservoirs in the chaotic time-series prediction and nonlinear channel equalization tasks because hybrid reservoirs possess the benefits of both parallel and deep configurations. The difference in performance may depend on the difficulty of the task. It would be worthy to investigate whether the performance of hybrid reservoirs is improved when other different tasks are applied.

We found that the use of two reservoirs provides the best performance in deep and hybrid configurations, and the use of multiple deep layers with more than two reservoirs does not improve the performance of the tasks investigated in the present study, unlike deep learning. The use of deeper layers compared with that of two reservoirs is a challenging issue in the further improvement of reservoir computing. In addition, the scalability of multiple reservoirs is crucial. To improve the scalability of multiple reservoirs, the use of a more sophisticated training algorithm, such as the augmented direct feedback alignment method [34], may be required. This method can be used to determine the connection weights between consecutive reservoirs without complicated calculations. Such a training method can help improve the performance of multiple reservoirs. We aim to investigate the effectiveness of a novel training method for multiple reservoirs in the future.

The configuration of parallel reservoirs may appear similar to an ensemble learning method that uses multiple reservoirs (learners) such as bagging and boosting. In ensemble learning, each reservoir is trained independently by using different datasets, and the final decision is made by considering the decision of a majority of multiple reservoirs. In contrast, our parallel reservoirs are trained using a single input with different masks, and the decision is made by using a weighted linear sum of all multiple reservoir nodes. Our parallel reservoirs may be simpler than the procedure of ensemble learning. It would be interesting to investigate whether the performance of parallel reservoirs can be improved by using ensemble learning techniques.

The feasibility of experimental implementation of this proposed scheme is another important issue. We have fabricated a single semiconductor laser with optical feedback on a photonic integrated circuit and demonstrated several tasks of reservoir computing in a previous study [16]. The implementation of multiple reservoir lasers on a single photonic chip is straightforward and technologically feasible. We aim to perform an experimental demonstration of compact reservoir computing on a photonic chip with multiple reservoirs in the future.

## 6 Conclusions

We investigated the feasibility of parallel and deep reservoir computing using semiconductor lasers with optical feedback to improve the performance of time-delayed all-optical reservoir computing. We proposed four reservoir configurations: single, parallel, deep, and hybrid reservoirs, and evaluated the quantitative performance of these four configurations in three tasks: chaotic time-series prediction task, nonlinear channel equalization task, and memory capacity measurement. Deep and hybrid configurations showed the best performance in the chaotic time-series prediction task, whereas parallel and hybrid configurations demonstrated the best performance in the nonlinear channel equalization task. There was minimal difference in the memory capacity among multiple reservoir configurations. The hybrid configuration showed the best performance for all three tasks. We also optimized the number of reservoirs when the total number of nodes was changed. The use of two reservoirs was suitable for deep and hybrid reservoirs in the chaotic time-series prediction task, whereas three or four reservoirs were suitable for parallel and hybrid reservoirs in the nonlinear channel equalization task.

The use of multiple reservoirs has great potential in improving the performance of reservoir computing in different processing tasks. A novel training technique can be applied for efficient learning of multiple reservoirs. Furthermore, the hybrid reservoir configuration can be used for high-performance edge computing.

## References

[1] Kitayama K., Notomi M., Naruse M. (2019). Novel Frontier of photonics for data processing—photonic accelerator. APL Photonics.

[2] Shen Y., Harris N. C., Skirlo S. (2017). Deep learning with coherent nanophotonic circuits. Nat. Photonics.

[3] Inagaki T., Haribara Y., Igarashi K. (2016). A coherent Ising machine for 2000-node optimization problems. Science.

[4] Naruse M., Berthel M., Drezet A. (2015). Single-photon decision maker. Sci. Rep..

[5] Larger L., Soriano M. C., Brunner D. (2012). Photonic information processing beyond turing: an optoelectronic implementation of reservoir computing. Opt. Express.

[6] Van der Sande G., Brunner D., Soriano M. C. (2017). Advances in photonic reservoir computing. Nanophotonics.

[7] Maass W., Natschläger T., Markram H. (2002). Real-time computing without stable states: a new framework for neural computation based on perturbations. Neural Comput..

[8] Jaeger H., Hass H. (2004). Harnessing nonlinearity: predicting chaotic systems and saving energy in wireless communication. Science.

[9] Tanaka G., Yamane T., Héroux J. B. (2019). Recent advances in physical reservoir computing: a review. Neural Netw..

[10] Bueno J., Maktoobi S., Froehly L. (2018). Reinforcement learning in a large-scale photonic recurrent neural network. Optica.

[11] Antonik P., Marsal N., Brunner D. (2019). Human action recognition with a large-scale brain-inspired photonic computer. Nat. Mach. Intell..

[12] Vandoorne K., Mechet P., Vaerenbergh T. V. (2014). Experimental demonstration of reservoir computing on a silicon photonics chip. Nat. Commun..

[13] Skalli A., Porte X., Haghighi N. (2022). Computational metrics and parameters of an injection-locked large area semiconductor laser for neural network computing. Opt. Mater. Express.

[14] Brunner D., Soriano M. C., Mirasso C. R. (2013). Parallel photonic information processing at gigabyte per second data rates using transient states. Nat. Commun..

[15] Nakayama J., Kanno K., Uchida A. (2016). Laser dynamical reservoir computing with consistency: an approach of a chaos mask signal. Opt. Express.

[16] Takano K., Sugano C., Inubushi M. (2018). Compact reservoir computing with a photonic integrated circuit. Opt. Express.

[17] Duport F., Schneider B., Semerieri A. (2012). All optical reservoir computing. Opt. Express.

[18] Paquot Y., Duport F., Smerieri A. (2012). Optoelectronic reservoir computing. Sci. Rep..

[19] Gallicchio C., Alessio M., Luca P. (2018). Comparison between DeepESNs and gated RNNs on multivariate time-series prediction. ..

[20] Sugano C., Kanno K., Uchida A. (2020). Reservoir computing using multiple lasers with feedback on a photonic integrated circuit. IEEE J. Sel. Top. Quantum Electron..

[21] Ortín S., Pesquera L. (2017). Reservoir computing with an ensemble of time-delay reservoirs. Cogn. Comput..

[22] Guo X. X., Xiang S. Y., Zhang Y. H. (2019). Four-channels reservoir computing based on polarization dynamics in mutually coupled VCSELs system. Opt. Express.

[23] Nguimdo R. M., Verschaffelt G., Danckaert J. (2015). Simultaneous computation of two independent tasks using reservoir computing based on a single photonic nonlinear node with optical feedback. IEEE Trans. Neural Netw. Learn. Syst..

[24] Zhong D., Yang H., Xi J. (2021). Predictive learning of multi-channel isochronal chaotic synchronization by utilizing parallel optical reservoir computers based on three laterally coupled semiconductor lasers with delay-time feedback. Opt. Express.

[25] Goldmann M., Köster F., Lüdge K., Yanchuk S. (2020). Deep time-delay reservoir computing: dynamics and memory capacity. Chaos.

[26] Freiberger M., Sackesyn S., Ma C. (2020). Improving time series recognition and prediction with networks and ensembles of passive photonic reservoirs. IEEE J. Sel. Top. Quantum Electron..

[27] Lang R., Kobayashi K. (1980). External optical feedback effects on semiconductor injection laser properties. IEEE J. Quantum Electron..

[28] Uchida A. (2012). *Optical Communication with Chaotic Lasers, Applications of Nonlinear Dynamics and Synchronization*.

[29] Kanno K., Uchida A., Bunsen M. (2016). Complexity and bandwidth enhancement in unidirectionally coupled semiconductor lasers with time-delayed optical feedback. Phys. Rev. E.

[30] Estébanez I., Fischer I., Soriano M. C. (2019). Constructive role of noise for high-quality replication of chaotic attractor dynamics using a hardware-based reservoir computer. Phys. Rev. Appl..

[31] Weigend A. S., Gershenfeld N. A. (1993). Results of the time series prediction competition at the Santa Fe Institute. Proc. IEEE Int. Conf. Neural Netw..

[32] Ortín S., Soriano M. C., Pesquera L. (2015). A unified framework for reservoir computing and extreme learning machines based on a single time-delayed neuron. Sci. Rep..

[33] Jaeger H. (2002). Short term memory in echo state network. *GMD Rep.*.

[34] Nakajima M., Inoue K., Tanaka K. (2022). Physical deep learning with biologically plausible training method. ..

